# Supplementary material for: Protein translation rate determines neocortical neuron fate
Source: Nat Commun. 2024 Jun 7;15:4879. doi: 10.1038/s41467-024-49198-w (PMC11161512; doi:10.1038/s41467-024-49198-w)
Supplement: Supplementary file 1 — Supplementary Information [file 41467_2024_49198_MOESM1_ESM.pdf]

Supplementary File

"Protein translation rate determines neocortical neuron fate"

Ekaterina Borisova, Andrew G. Newman, Marta Couce Iglesias, Rike Dannenberg, Theres Schaub, Bo Qin, Alexandra Rusanova, Marisa Brockmann, Janina Koch, Marieatou Daniels, Paul Turko, Olaf Jahn, David R. Kaplan, Marta Rosário, Takao Iwawaki, Christian M. T. Spahn, Christian Rosenmund, David Meierhofer, Matthew L. Kraushar, Victor Tarabykin, Mateusz C. Ambrozkiewicz

Contents:

1. Supplementary Figures and Figure Legends

SUPPLEMENTARY FIGURES AND FIGURE LEGENDS

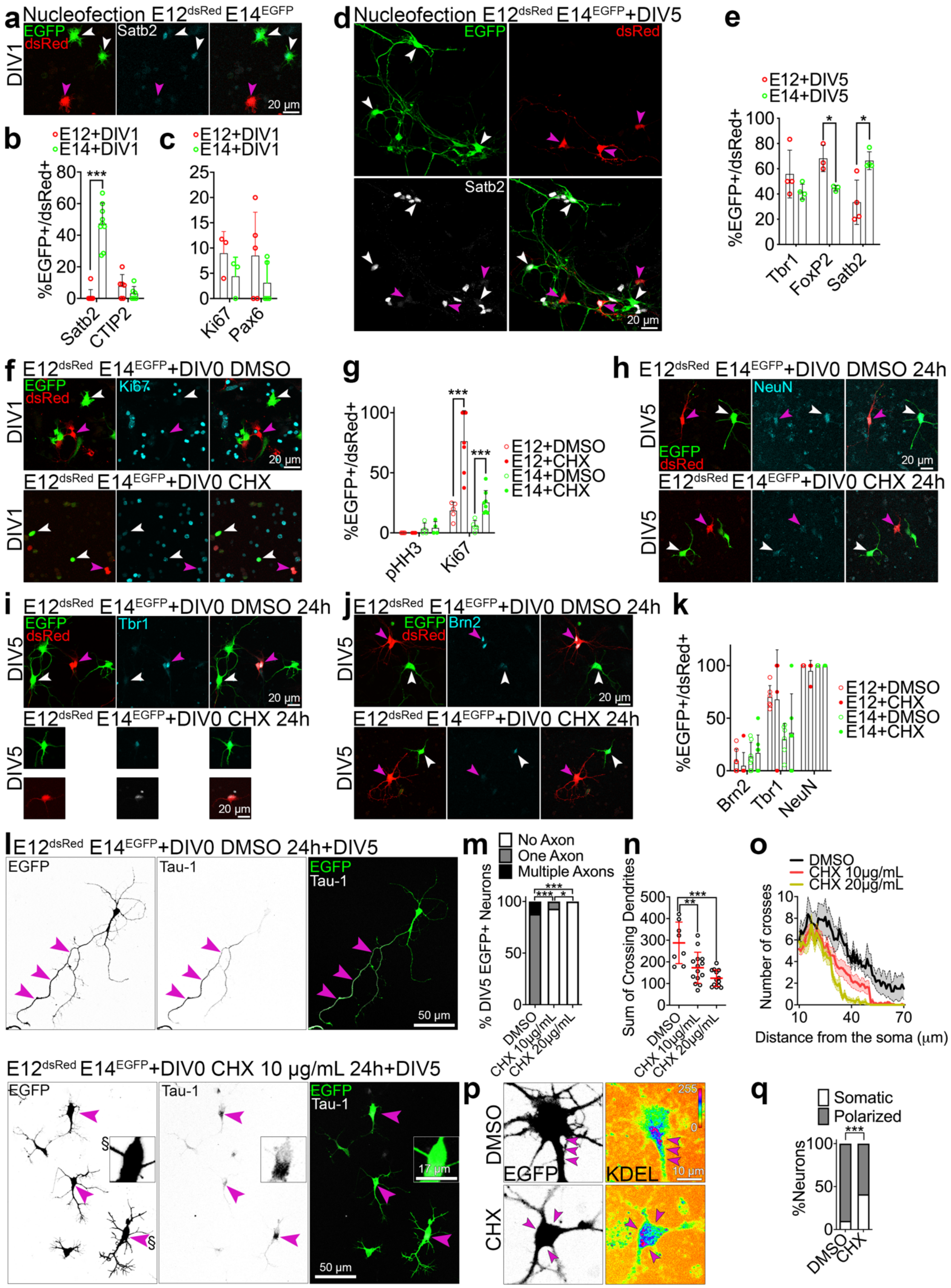

Fig. S1. Related to Fig. 1. Transient translation inhibition alters the cell cycle dynamics of precursor cells and disrupts neuritogenesis in cortical neurons.
(a, d) Images of primary DIV1 (a) or DIV5 (d) neurons immunolabeled for EGFP, dsRed, and Satb2. Cortical cells from E12 embryo were nucleofected to express dsRed (purple arrowheads), and cortical cells from E14 embryo to express EGFP (white arrowheads). Both cell populations were mixed together and plated on a single glass coverslip. (b, c, e) Quantification of the cell identity in DIV1 (b-c) or DIV5 neurons (e) derived from E12 or E14 cortex. (f-q) Images of immunolabeled primary cells prepared as described in the legend of Fig. 1. Cells were fixed at DIV1 at the end of treatment (f, g) of at DIV5 (h, i, j, l, p) and immunolabeled for indicated marker proteins. (g, k) Quantification of cellular fates. (l) Purple arrowheads indicate enrichments of Tau-1. The neuron marked with a paragraph symbol is magnified on the bottom panel. (m) Quantification of neuronal polarity. Axon was defined as the neurite with prominent Tau-1 enrichment. (n, o) Quantification of dendritic complexity in DMSO- and CHX-treated DIV5 neurons. (q) Quantification of KDEL signal polarity. Single dots on graphs represent data points. Bar graphs and error bars indicate mean ± S.D. Red line and error bars on (n) indicate mean ± S.D. Results on (o) are represented as averages ± S.E.M. For statistical analyses, D'Agostino and Pearson normality test and (b, c, e, g), unpaired t-test or Mann-Whitney test (Supplementary Data S1); (m), Chi-square test; (k), Kruskal-Wallis test with Dunn's post hoc test; (n), one-way ANOVA with Bonferroni post-hoc test; (q), two-sided Fisher's exact test. *** $p < 0.001$; $0.001 <$ ** $p < 0.01$; $0.01 <$ * $p < 0.05$.

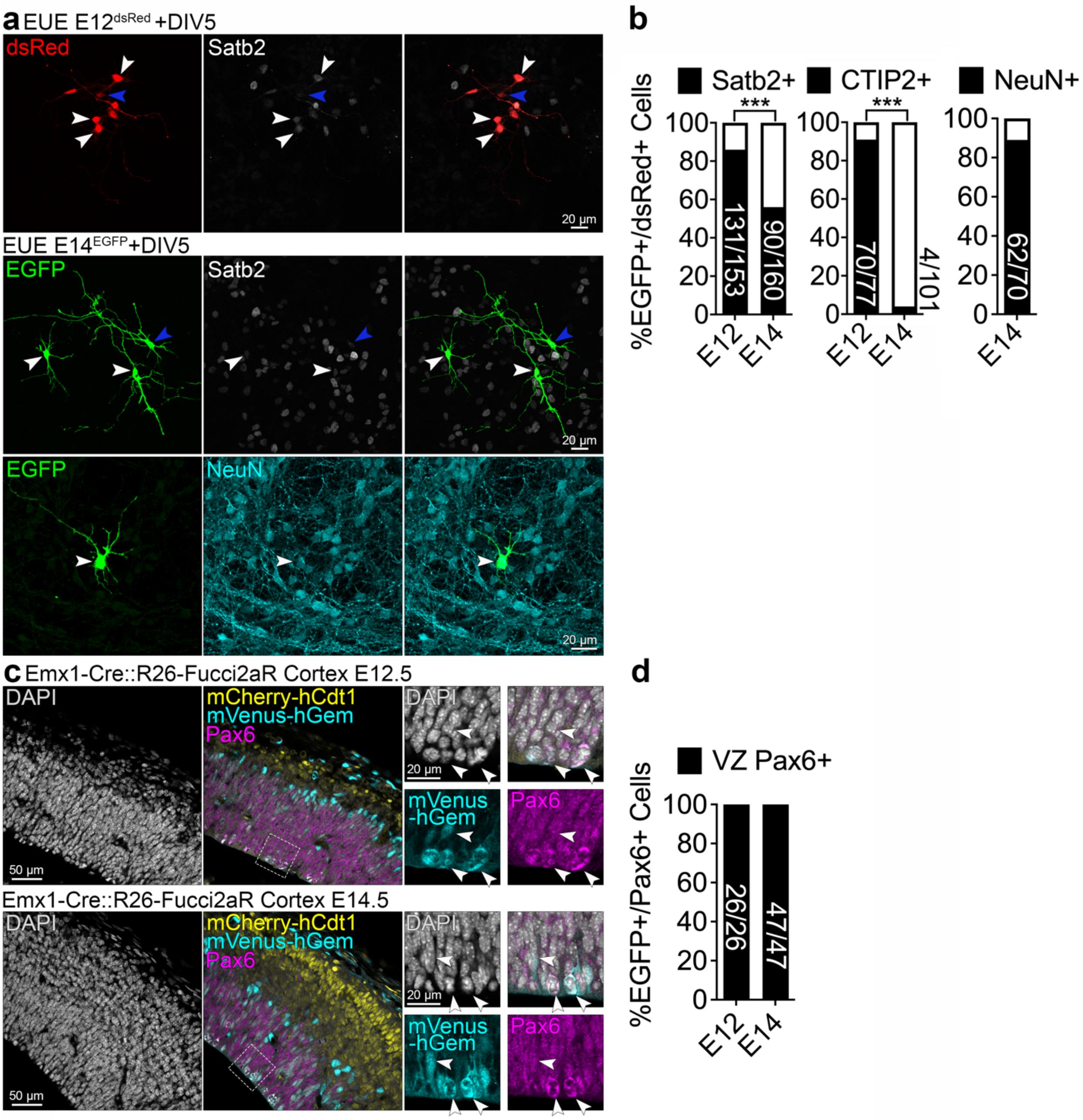

Fig. S2. Related to Fig. 2 and Fig. 3. Combinatorial expression of marker proteins is a characteristic of early and later-born cortical neurons.

(a) E12 and E14-derived cells, prepared as described in the legend to Fig. 2, were fixed at DIV5 and immunolabeled for Satb2 and NeuN. White arrowheads point to neurons expressing Satb2, blue ones indicate example neurons with lower or no Satb2 expression. (b) Quantification of neuronal fates. (c) Representative images of E12.5 and E14.5 cortical coronal section of *Emx1*$^{Cre/+}$; Fucci2aR, immunolabeled for Venus, mCherry, and Pax6. The dotted squares indicate represented regions of interest. White arrows point to mVenus-positive Pax6-positive ventricular progenitors, quantified in (d). For statistics, two-sided Fisher's exact test. *** P < 0.001. Numbers indicate the cell numbers from three independent cultures used to quantify the proportion of positive neurons.

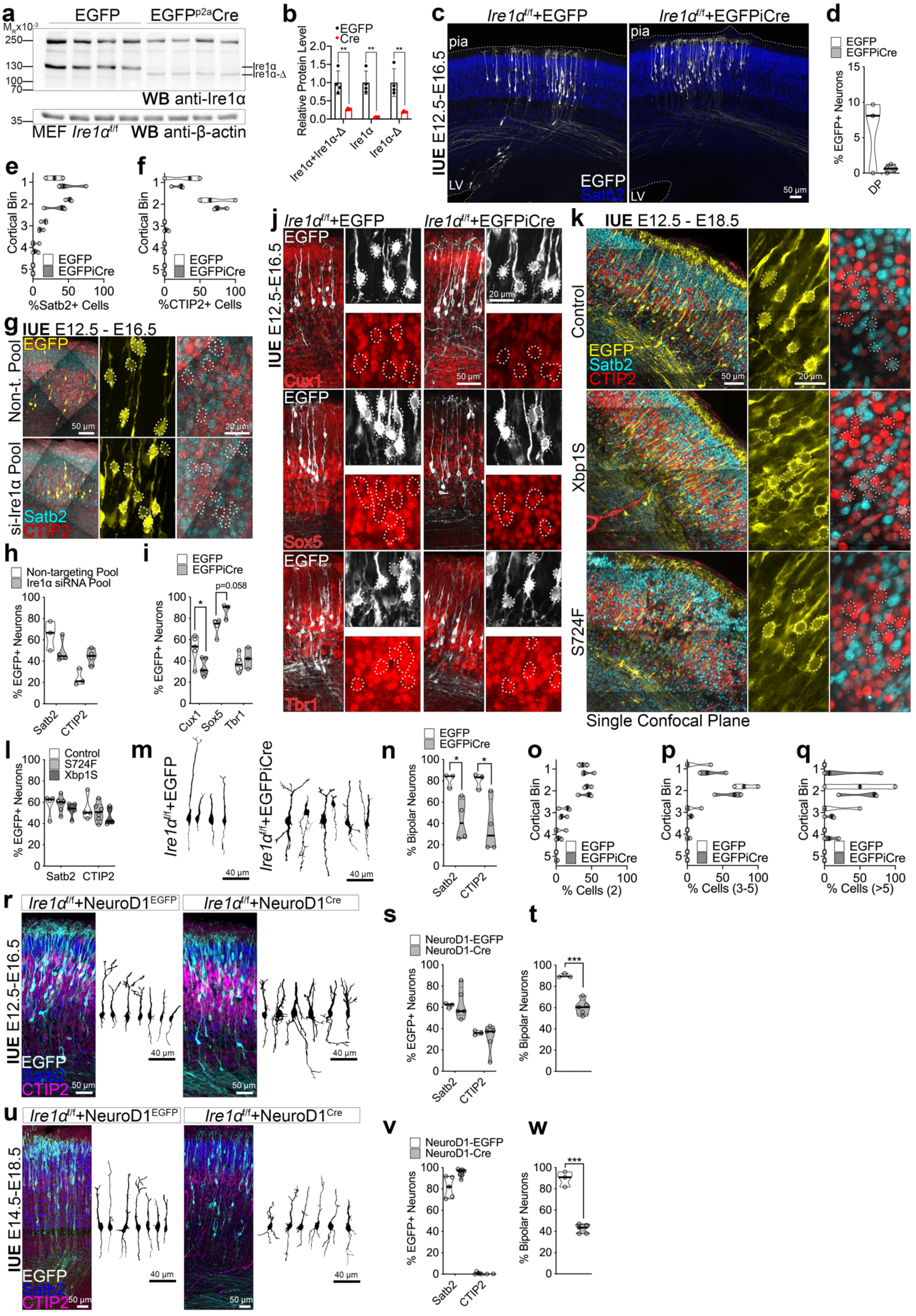

a
EGFP
EGFPp2aCre
Ire1α
Ire1α-Δ
WB anti-Ire1α
MEF Ire1α f/f
WB anti-β-actin
b
EGFP
Cre
Relative Protein Level
Ire1α+Ire1α-Δ
Ire1α
Ire1α-Δ
c
Ire1α f/f+EGFP
Ire1α f/f+EGFPiCre
IUE E12.5-E16.5
pia
LV
EGFP
Satb2
50 µm
d
EGFP
EGFPiCre
% EGFP+ Neurons
DP
e
Cortical Bin
%Satb2+ Cells
f
%CTIP2+ Cells
g
IUE E12.5 - E16.5
Non-t. Pool
si-Ire1α Pool
h
Non-targeting Pool
Ire1α siRNA Pool
% EGFP+ Neurons
Satb2
CTIP2
i
p=0.058
Cux1
Sox5
Tbr1
j
IUE E12.5-E16.5
k
IUE E12.5 - E18.5
Control
Xbp1S
S724F
Single Confocal Plane
l
Control
S724F
Xbp1S
m
n
% Bipolar Neurons
o
% Cells (2)
p
% Cells (3-5)
q
% Cells (>5)
r
Ire1α f/f+NeuroD1EGFP
Ire1α f/f+NeuroD1Cre
s
NeuroD1-EGFP
NeuroD1-Cre
t
u
IUE E14.5-E18.5
v
w

Fig. S3. Related to Fig. 5. Ire1α-driven acquisition of the Satb2 identity, but not of bipolar morphology, is embedded in neuronal progenitors.

(a) Representative Western blotting and quantification (b) in DIV5 *Ire1α*$^{f/f}$ murine embryonic fibroblasts (MEF) infected with AAV particles encoding indicated constructs. (c) Representative image for Fig. 5n, depicting the lateral ventricle (LV). (d) Quantification of Satb2- and CTIP2-double positive neurons after IUE described for Fig. 5n. (e-f) Laminar positioning of EGFP-positive neurons expressing Satb2 (e) or CTIP2 (f) in the cortex after IUE described for Fig. 5n. The number of neurons in each cortical bin was normalized to the total number of electroporated neurons. (g-h) Representative images and neuronal fate quantification after IUE in E12.5 wild-type cortex using control siRNA or siRNA targeting endogenous Ire1α. (i-j) Quantification of neuronal fates and representative images after IUE described for Fig. 5n. (k) Representative images of immunostaining against EGFP, Satb2 and CTIP2 in E18.5 coronal cortical sections of wild-type embryos after IUE at E12.5 with plasmids encoding for EGFP and mock DNA, Xbp1S, or human IRE1α S724F mutant. (l) Quantification of neuronal fates after IUE described in (k). (m) Example EGFP-based tracings of single neurons after IUE in Fig. 5n. (n) Quantification of the proportion of bipolar neurons expressing Satb2 or CTIP2 after IUE described in Fig. 5n. (o-q) Laminar positioning of EGFP-positive neurons after IUE described for Fig. 5n with 2 (o), 3-5 (p), or more than 5 (q) principal neurites emanating from the soma. (r, u) Representative images of immunostaining in coronal cortical sections from E16.5 (r) or E18.5 (u) *Ire1α*$^{f/f}$ embryos after IUE at E12.5 (r) or E14.5 (u) with plasmids encoding for EGFP under the promoter of *NeuroD1* gene or Cre under the promoter of *NeuroD1* together with loxP-Stop-loxP-EGFP. Shown are also representative, EGFP-based tracings of single neurons. (s, v) Quantification of neuronal cell identity in (r) and (u). (t, w) Average proportion of bipolar neurons in (r) and (u). Bar graphs show averages ± S.D. Violin plots indicate individual data points, thick line median and thin lines quartiles. For statistical analyses, (b, d, h-i, n, s-t, v-w), D'Agostino-Pearson and Kolmogorov-Smirnov normality test, Mann-Whitney, or unpaired t-test, Welch's correction (Supplementary Data S1); (e-f, l, o-q), two-way ANOVA with Šidák correction. *** $p < 0.001$; $0.001 <$ ** $p < 0.01$; $0.01 <$ * $p < 0.05$.

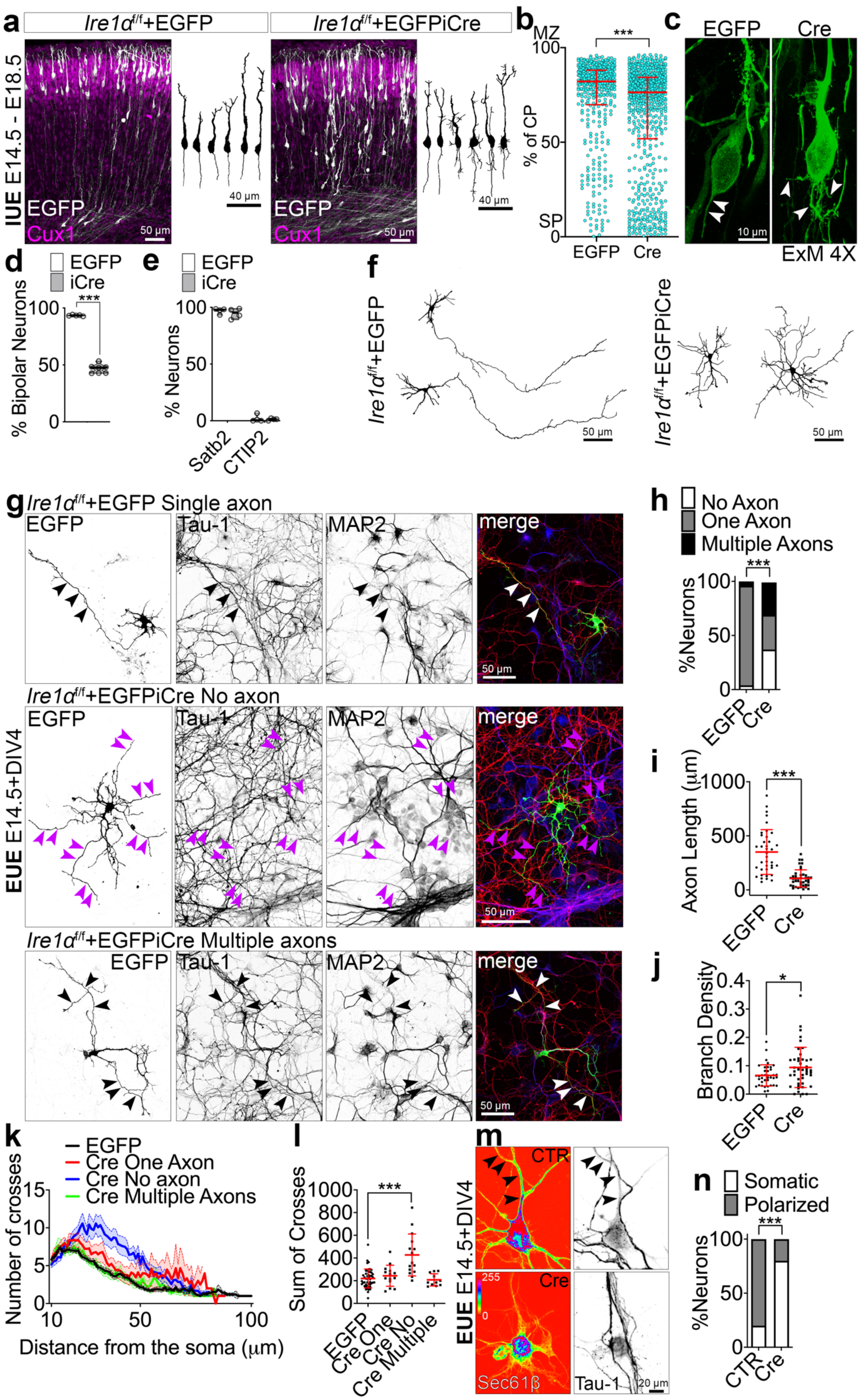

a
Ire1α f/f+EGFP
Ire1α f/f+EGFPiCre
IUE E14.5 - E18.5
EGFP
Cux1
50 µm
40 µm
b
MZ
SP
% of CP
***
EGFP
Cre
c
EGFP
Cre
10 µm
ExM 4X
d
EGFP
iCre
% Bipolar Neurons
e
% Neurons
Satb2
CTIP2
f
Ire1α f/f+EGFP
Ire1α f/f+EGFPiCre
50 µm
g
Ire1α f/f+EGFP Single axon
Ire1α f/f+EGFPiCre No axon
Ire1α f/f+EGFPiCre Multiple axons
EUE E14.5+DIV4
EGFP
Tau-1
MAP2
merge
h
No Axon
One Axon
Multiple Axons
%Neurons
i
Axon Length (µm)
j
Branch Density
k
EGFP
Cre One Axon
Cre No axon
Cre Multiple Axons
Number of crosses
Distance from the soma (µm)
l
Sum of Crosses
EGFP
Cre One
Cre No
Cre Multiple
m
EUE E14.5+DIV4
CTR
Cre
Sec61β
Tau-1
20 µm
n
Somatic
Polarized
%Neurons
CTR
Cre

Fig. S4. Related to Fig. 5. Ire1α is indispensable for bipolar morphology, axon specification and proper laminar positioning of later-born upper layer neurons.
(a) Representative images of EGFP and upper layer-expressed Cux1 immunostaining in coronal cortical sections from e18.5 *Ire1α*$^{f/f}$ embryos after IUE at e14.5 with indicated plasmids and semi-automatic, EGFP-fluorescence based tracings of single neurons. (b) Laminar distribution of cortical neurons in e18.5 *Ire1α*$^{f/f}$ brains after IUE described in (a). The position of each neuron was normalized to the thickness of the CP and represented as a dot. 0% - Subplate (SP), 100% - Marginal Zone (MZ). Graph contains pooled data from indicated number of brains (Supplementary Data S1). (c) Representative images of EGFP immunostaining after expansion microscopy (ExM) in cortical sections from (a). Arrowheads point to trailing processes. (d) Average proportion of bipolar neurons from (a). (e) Quantification of neuronal identities in experiment in (a). (f) Representative EGFP-based tracings of *Ire1α*$^{f/f}$ neurons at DIV4 after EUE to express EGFP or EGFP and Cre. (g) Neurons in (f) were fixed and immunostained with axonal or dendritic markers. White and black arrowheads indicate axons, purple ones indicate neurites lacking Tau-1 expression. (h) The number of axons projected from a single neuron. Average axon length (i), average axon branch density per micrometer (j), Sholl analysis diagram (k) and average sum of crossing dendrites (l) in neurons expressing EGFP or EGFP and Cre, projecting no, one, or multiple axons. (m) Representative EGFP signals and Tau-1 immunostaining of *Ire1α*$^{f/f}$ DIV4 neurons after EUE to co-express EGFP-Sec61β and empty vector (CTR) or Cre. (n) Quantification of EGFP-Sec61β localization in experiment in (m). Line and error bars on (b) indicate median and interquartile range, scatter plots in (i), (j), (l) and violin plots on (d) and (e) show individual data points, thick lines are median and thin lines - quartiles. Results on (k) are represented as averages ± S.E.M. Statistics for (b), (d-e), (i-j), D'Agostino-Pearson normality test and Mann-Whitney or unpaired t-test (Supplementary Data S1); (h), Chi-square test; (l), Kruskal-Wallis with Dunn's multiple comparisons test; (n), two-sided Fisher's exact test. *** $p < 0.001$; $0.01 <$ * $p < 0.05$.

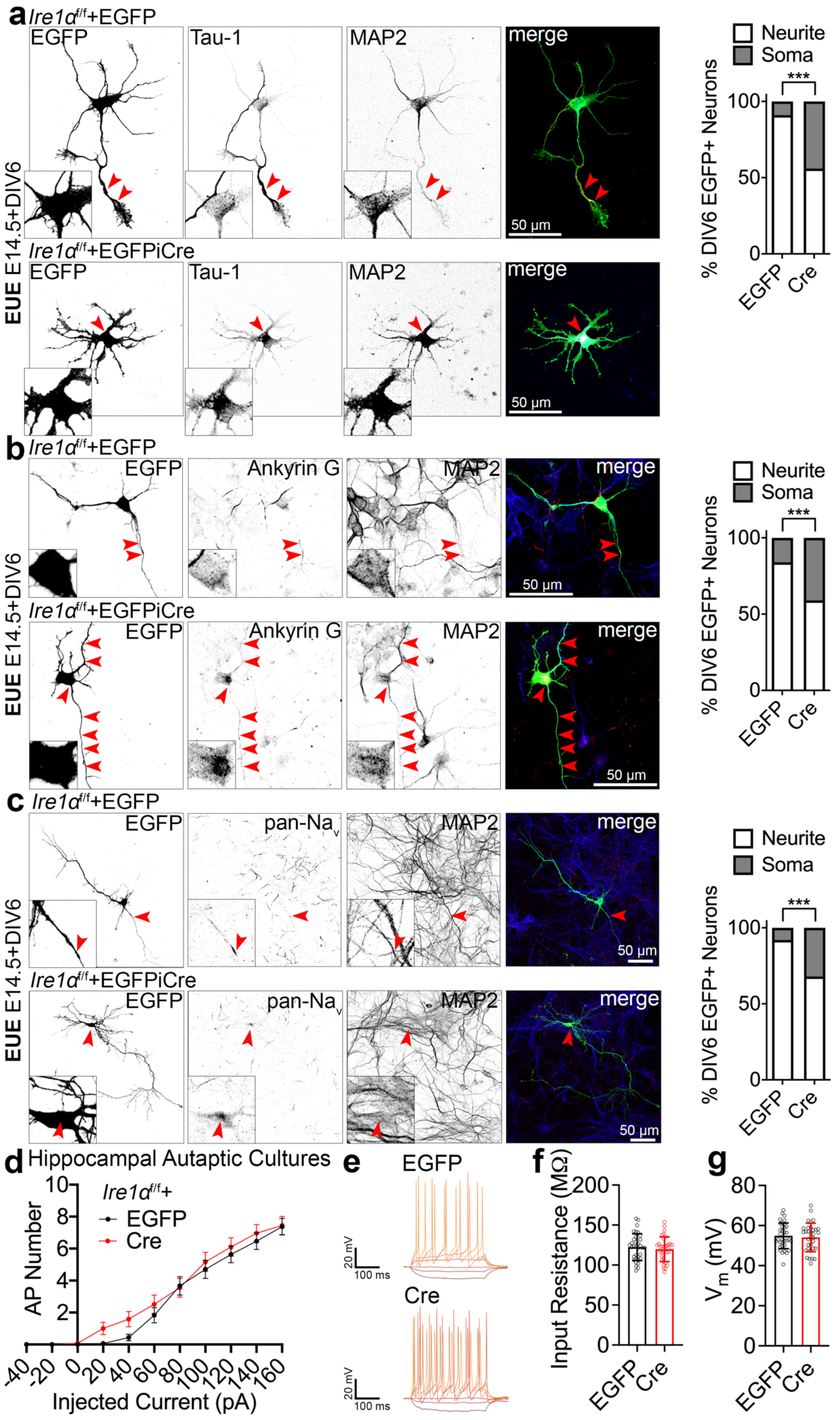

a Ire1α^f/f+EGFP
EGFP
Tau-1
MAP2
merge
50 µm
Ire1α^f/f+EGFPiCre
EUE E14.5+DIV6
Neurite
Soma
***
% DIV6 EGFP+ Neurons
EGFP
Cre
b Ire1α^f/f+EGFP
Ankyrin G
c Ire1α^f/f+EGFP
pan-Na_V
d Hippocampal Autaptic Cultures
AP Number
Injected Current (pA)
e EGFP
Cre
20 mV
100 ms
f Input Resistance (MΩ)
g V_m (mV)

Fig. S5. Related to Fig. 5. Loss of *Ire1α* disrupts axon initial segment and current responses.

(a-c) Images of representative DIV6 primary cortical *Ire1α*$^{f/f}$ neurons after EUE at E14.5 for EGFP or EGFP and Cre expression. Neuronal cultures were immunolabeled for indicated axonal and dendritic marker proteins. Graphs show quantification of the subcellular distribution of axonal markers. *Ire1α*-deficient neurons display somatic accumulations of Tau-1 (a), Ankyrin G (b), and Na_v (c). Red arrowheads indicate enrichment of axonal markers. Insets on the bottom left of black and white images are zoom-ins to the somata or places of axonal marker accumulation. (d-g) Electrophysiological recordings from autaptic hippocampal cultures prepared from P0 *Ire1α*$^{f/f}$ pups. Neuronal cultures were infected with lentiviruses encoding for EGFP or Cre. (d) Quantification of the number of APs generated after injection of increasing amount of current. (e) Example traces of recordings described in (d). Average input resistance (f) and holding potential (g). Data points and error bars on (d) represent mean ± S.E.M. and graphs on (f-g), individual data points, mean ± S.D. For statistical analyses, (a-c), two-sided Fisher's exact test; (d), one-way ANOVA; (f-g), Mann-Whitney test. *** $p < 0.001$.

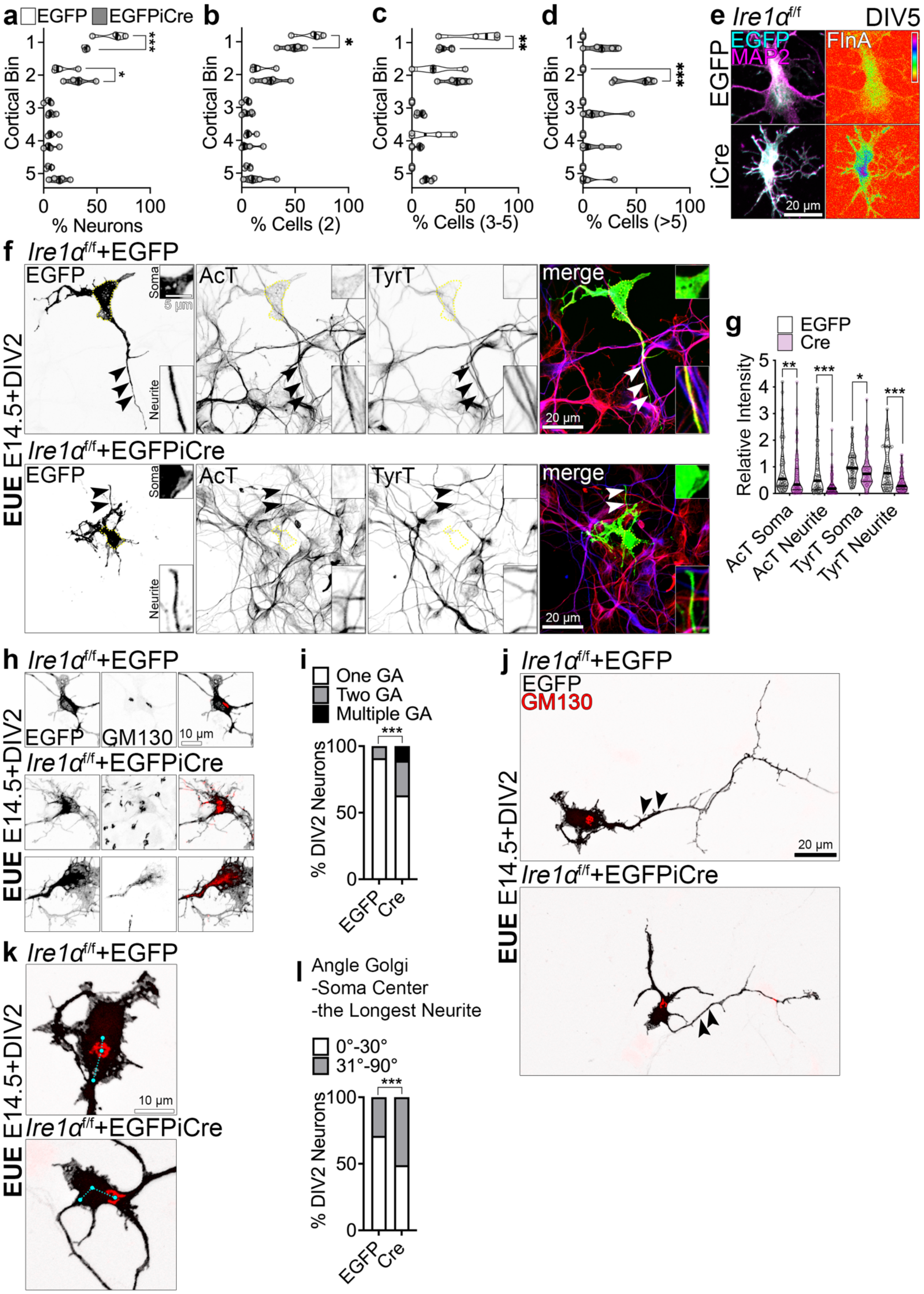

a EGFP EGFPiCre
Cortical Bin
% Neurons
b
% Cells (2)
c
% Cells (3-5)
d
% Cells (>5)
e Ire1α f/f DIV5
EGFP
MAP2
FlnA
iCre
20 µm
f Ire1α f/f+EGFP
EGFP
Soma
5 µm
Neurite
AcT
TyrT
merge
20 µm
Ire1α f/f+EGFPiCre
EUE E14.5+DIV2
g
EGFP
Cre
Relative Intensity
AcT Soma
AcT Neurite
TyrT Soma
TyrT Neurite
h Ire1α f/f+EGFP
GM130
10 µm
Ire1α f/f+EGFPiCre
i
One GA
Two GA
Multiple GA
% DIV2 Neurons
j Ire1α f/f+EGFP
k Ire1α f/f+EGFP
l Angle Golgi
-Soma Center
-the Longest Neurite
0°-30°
31°-90°

Fig. S6. Related to Fig. 5. Loss of *Ire1α* disrupts microtubule stability and Golgi apparatus polarity.

(a-d) Laminar positioning of EGFP-positive neurons projecting 2 (b), 3-5 (c), and more than 5 (d) principal neurites emanating from the soma after IUE described in the S4a figure legend. (e) Representative images of DIV5 *Ire1α*$^{f/f}$ neurons after EUE at E14.5 to express indicated constructs immunolabeled against Filamin A. (f-l) Representative images of DIV2 *Ire1α*$^{f/f}$ neurons after EUE at E14.5 to express indicated constructs immunolabeled against indicated marker proteins. Arrowheads on (f) and (j) point to the longest neurite. Acetylated tubulin, AcT; tyrosinated tubulin, TyrT. (g) Quantification of the fluorescence intensity of the indicated form of tubulin in (f). (i) Quantification of the number of Golgi apparatus (GA) found in EGFP- or EGFP- and Cre- expressing DIV2 *Ire1α*$^{f/f}$ neurons. (k-l) Representative images and quantification (l) of the Golgi apparatus position relative to the center of the soma and the longest neurite. For statistics, (a-d), two-way ANOVA with Šidák correction; (g), D'Agostino-Pearson normality test and unpaired t-test or Mann-Whitney test (Supplementary Data S1); (i) and (l), two-sided Fisher's exact test. *** $p < 0.001$; $0.001 <$ ** $p < 0.01$; $0.01 <$ * $p < 0.05$.

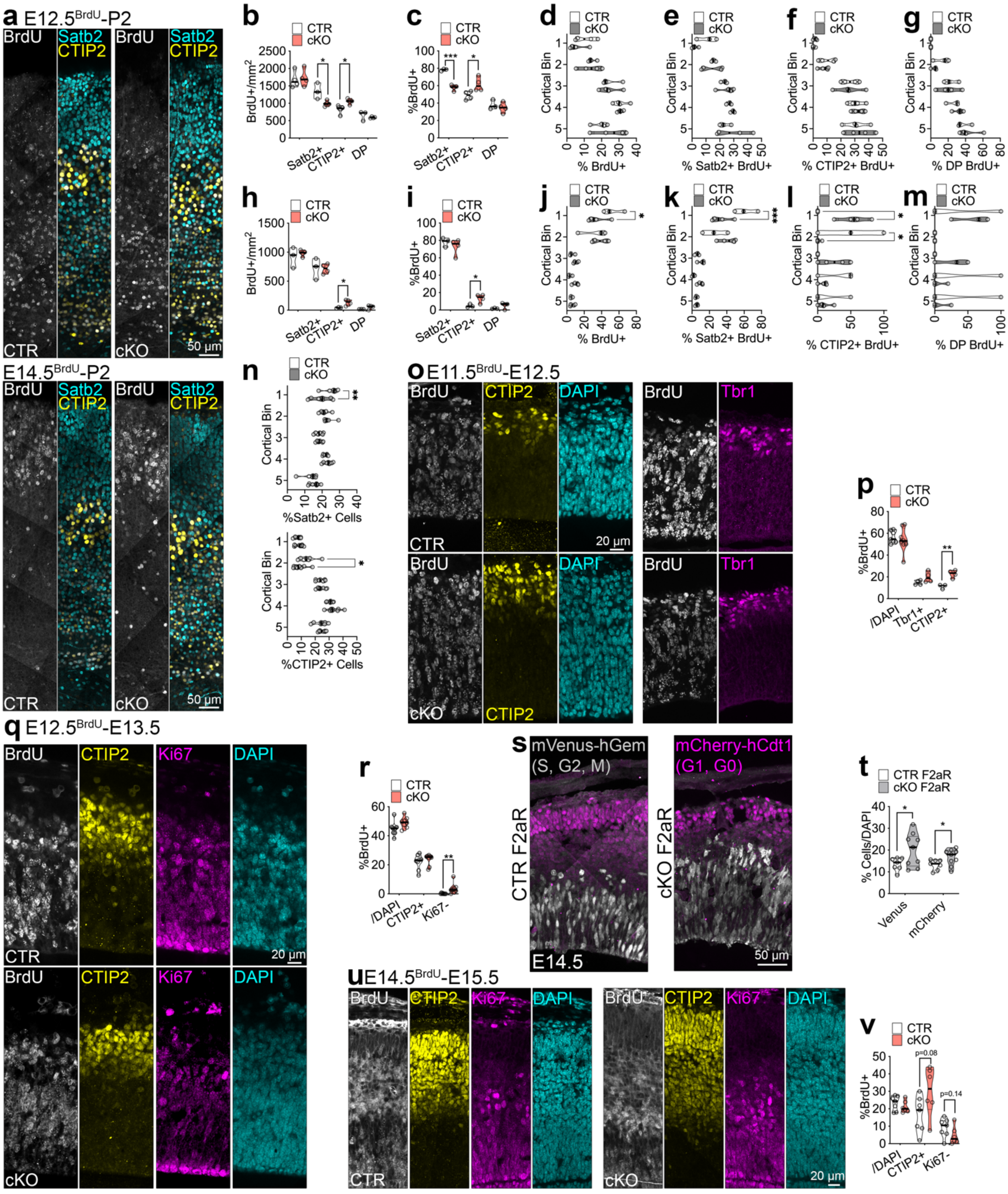

Fig. S7. Related to Fig. 5. Birth dates and laminar positions of Satb2+ and CTIP2+ neurons in *Ire1α* cKO cortex.

(a) Representative images of immunostaining in P2 control or cKO coronal cortical sections after BrdU pulse at E12.5 or E14.5. (b), (h) Quantification of cell density expressing an indicated nuclear stain. DP, Satb2- and CTIP2-double positive. (c), (i) Quantification of the proportion of neurons expressing indicated marker within all BrdU+ cells. (d-g), (j-m) Laminar distribution of BrdU-positive neurons expressing indicated marker. The proportion of neurons

in each bin was normalized to the total number of neurons of a given type within the CP. (n) Laminar distribution of Satb2- and CTIP2-positive neurons in CTR and cKO P2 cortex. (o, q, u) Representative images of immunostaining in control or cKO coronal cortical sections after BrdU pulse at E11.5 (o), E12.5 (q), or E14.5 (u). (p), (r), (v) Quantifications of the proportions of cells expressing indicated markers 24h after the pulse at E12.5 (p), E13.5 (r) and E15.5 (v). (s) Representative images of E14.5 cortical coronal section of *Ire1α*$^{f/+}$; *Emx1*$^{Cre/+}$; Fucci2aR (CTR F2aR) or *Ire1α*$^{f/f}$; *Emx1*$^{Cre/+}$; Fucci2aR (cKO F2aR), immunolabeled for Venus and mCherry. (t) Quantification of the proportion of cells expressing indicated fluorophore normalized to DAPI count. Violin plots indicate individual data points, thick line shows median and thin lines indicate quartiles. For statistical analyses, D'Agostino-Pearson normality test and (b-c), (h-i), (p), (r), (t), (v), unpaired t-test or Mann-Whitney test (Supplementary Data S1); (d-g) and (j-n), two-way ANOVA with Šidák correction. *** $p < 0.001$; $0.001 <$ ** $p < 0.01$; $0.01 <$ * $p < 0.05$.

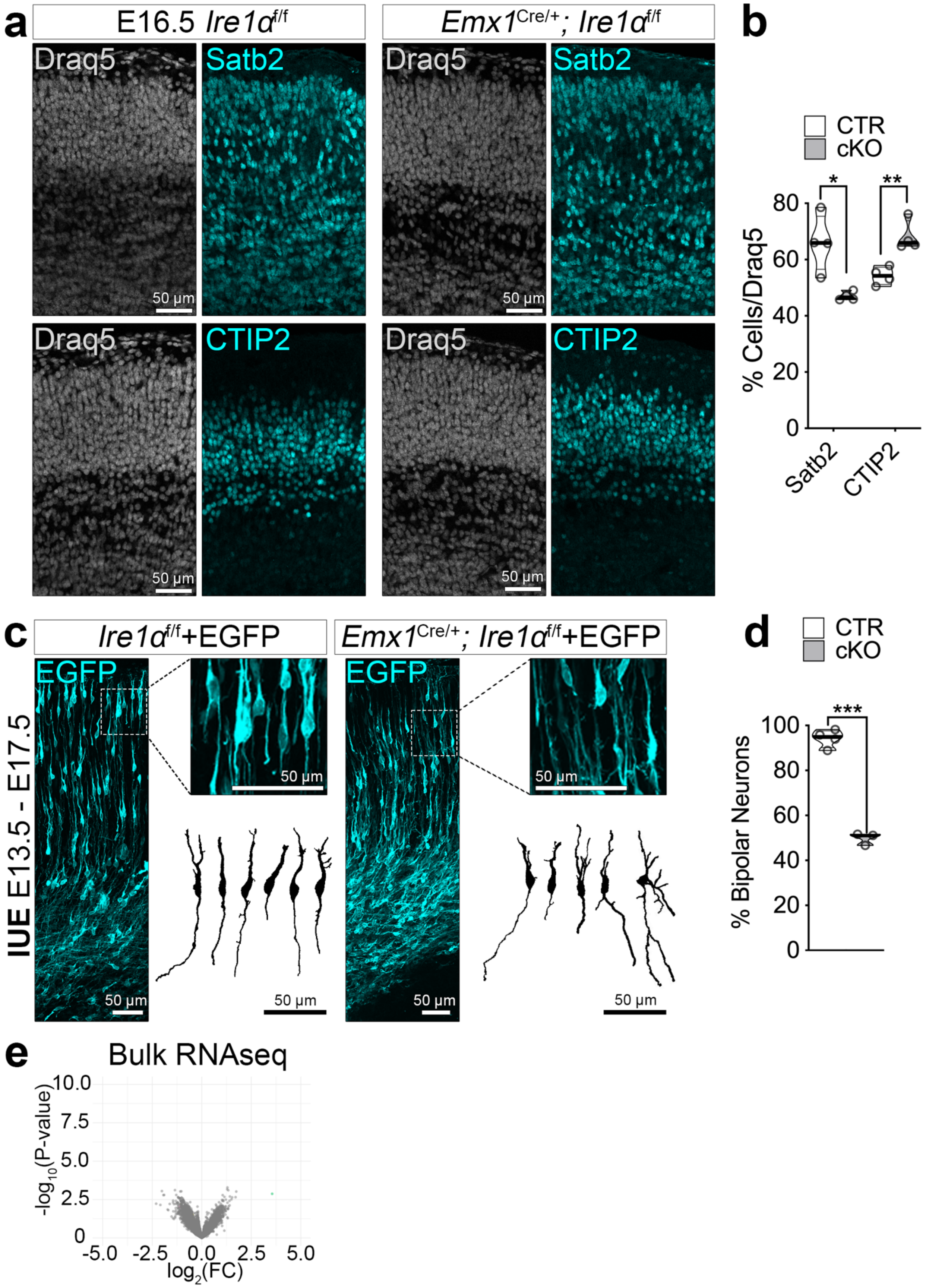
a
E16.5 Ire1α f/f
Emx1 Cre/+; Ire1α f/f
Draq5
Satb2
CTIP2
50 µm
b
CTR
cKO
*
**
80
60
40
20
0
% Cells/Draq5
Satb2
CTIP2
c
Ire1α f/f+EGFP
Emx1 Cre/+; Ire1α f/f+EGFP
EGFP
IUE E13.5 - E17.5
50 µm
d
CTR
cKO
***
100
80
60
40
20
0
% Bipolar Neurons
e
Bulk RNAseq
10.0
7.5
5.0
2.5
0
-log10(P-value)
-5.0
-2.5
0.0
2.5
5.0
log2(FC)

Fig. S8. Related to Fig. 5 and Fig. 6. Aberrant neuronal identity in forebrain-specific Ire1α knock-out mice. (a) Representative images of E16.5 cortical coronal sections immunolabeled for Satb2 and CTIP2 and stained for Draq5 from control (*Ire1α*$^{f/f}$) and *Ire1α* cKO (*Emx1*$^{Cre/+}$; *Ire1α*$^{f/f}$) mice. (b) Quantification of proportion of Satb2- and CTIP2-expressing cortical plate cells normalized to Draq5. (c) Representative images of E17.5 cortical coronal sections after IUE at E13.5 with EGFP-expressing plasmid, immunolabeled for EGFP and stained for DAPI from control (*Ire1α*$^{f/f}$) and *Ire1α* cKO (*Emx1*$^{Cre/+}$; *Ire1α*$^{f/f}$) mice. Dotted square indicates the represented region of interest and example EGFP-based tracings of neuronal morphologies. (d) Quantification of bipolar neuronal morphologies in control and cKO cortex. (e) Volcano plots for *Ire1α* cKO RNAseq in bulk tissue. FC; fold change. All P-values are Benjamini-Hochburg adjusted p-values. Violin plots on (b) and (d) show individual data points, thick lines are median and thin lines – quartiles. Statistics for (b) and (d), unpaired t-test with Welch's correction. *** $p < 0.001$; $0.001 <$ ** $p < 0.01$; $0.01 <$ * $p < 0.05$.

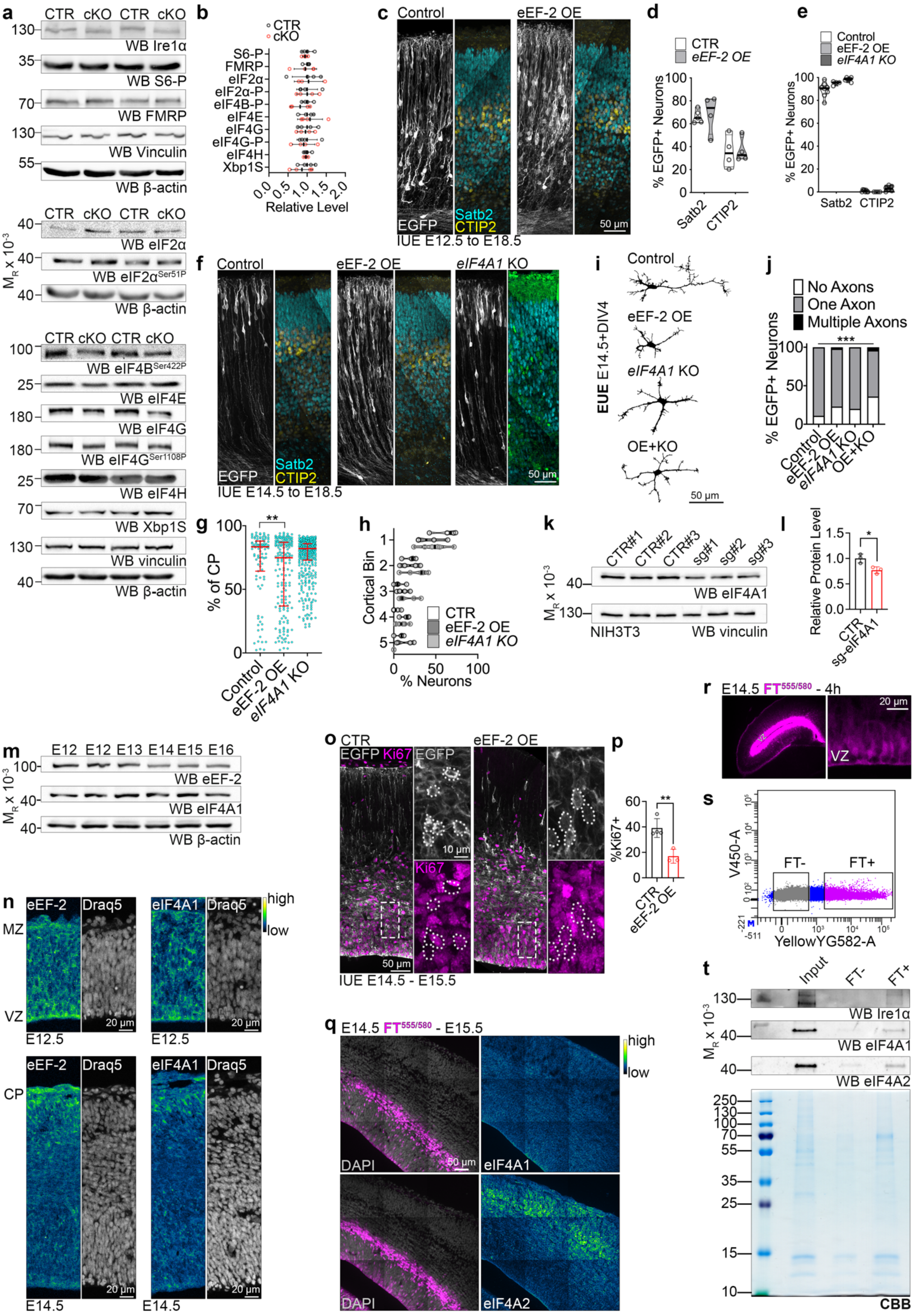
a
CTR cKO CTR cKO
WB Ire1α
WB S6-P
WB FMRP
WB Vinculin
WB β-actin
WB eIF2α
WB eIF2αSer51P
WB eIF4BSer422P
WB eIF4E
WB eIF4G
WB eIF4GSer1108P
WB eIF4H
WB Xbp1S
WB vinculin
MR x 10-3
b
CTR
cKO
S6-P
FMRP
eIF2α
eIF2α-P
eIF4B-P
eIF4E
eIF4G
eIF4G-P
eIF4H
Xbp1S
Relative Level
c
Control
eEF-2 OE
EGFP
Satb2
CTIP2
50 µm
IUE E12.5 to E18.5
d
CTR
eEF-2 OE
% EGFP+ Neurons
Satb2
CTIP2
e
Control
eEF-2 OE
eIF4A1 KO
f
Control
eEF-2 OE
eIF4A1 KO
IUE E14.5 to E18.5
g
% of CP
h
Cortical Bin
% Neurons
i
EUE E14.5+DIV4
OE+KO
j
No Axons
One Axon
Multiple Axons
k
CTR#1 CTR#2 CTR#3 sg#1 sg#2 sg#3
WB eIF4A1
NIH3T3
l
Relative Protein Level
sg-eIF4A1
m
E12 E12 E13 E14 E15 E16
WB eEF-2
n
eEF-2
Draq5
eIF4A1
high
low
MZ
VZ
CP
E12.5
E14.5
20 µm
o
Ki67
10 µm
IUE E14.5 - E15.5
p
%Ki67+
q
E14.5 FT555/580 - E15.5
DAPI
eIF4A2
r
E14.5 FT555/580 - 4h
s
V450-A
FT-
FT+
YellowYG582-A
t
Input
WB eIF4A2
CBB

Fig. S9. Related to Fig. 7. Ire1α-mediated protein translation regulation in the developing cortex specifically involves eIF4A1.

(a) Representative results of Western blotting in E18.5 cortical lysates from control and *Ire1α* cKO using indicated antibodies. (b) The expression level of analyzed protein was normalized to the amount of beta-actin in the sample and expressed as relative protein level. (c-h) Representative images (stitched tiles) of EGFP fluorescence signals and immunolabeling for Satb2 and CTIP in E18.5 brains of wild-type embryos after IUE at E12.5 (c), or E14.5 (f) with plasmids encoding for EGFP (Control) or for EGFP and eEF-2 (eEF-2 OE), or for EGFP and eIF4A1 gRNAs and Cas9 nickase (*eIF4A1* KO). Quantification of neuronal identity (d-e) and laminar positioning of neurons within the cortical plate (g-h). Graph contains pooled data from indicated number of brains (Supplementary Data S1) (i-j) The number of axons projected from a single neuron at DIV4 after EUE to achieve indicated genotypes. Neurons were fixed and immunolabeled from axonal and dendritic markers. OE+KO, simultaneous eEF-2 OE and eIF4A1 KO. (i) Representative EGFP-based tracings of DIV4 neurons. Apart from Control, shown are neurons with 0 axons. (k-l) Representative Western blotting and quantification of the efficiency of sgRNA targeting eIF4A1 16 hours post transfection of NIH3T3 cells. (m) Representative expression pattern of indicated proteins across development of the cortex. (n) Representative images of the immunostaining in the coronal cortical slice for indicated proteins and Draq5, a nuclear marker. VZ, ventricular zone; MZ, marginal zone; CP, cortical plate. (o) Representative images of EGFP fluorescence signals and immunolabeling for Ki67 24 hours after IUE at E14.5 in wild-type cortex. (p) Quantification of the fraction of EGFP-positive neurons expressing Ki67 from the experiment in (o). (q) Representative immunostaining in coronal cortical section using indicated antibodies at E15.5 after FlashTag pulse at E14.5. (r-t) FlashTag- and FACS-based isolation of apical progenitors from the developing cortex and the results of Western blotting using indicated antibodies in FlashTag-positive (FT+) progenitors. Coomassie brilliant blue, CBB. Bar graphs indicate mean ± S.D. Violin plots represent single data points, thick line median and thin lines quartiles. Line and error bars on (g) indicate median and interquartile range and scatter plots individual positions of single neurons. For statistics on (b) unpaired t-test with post-hoc Holm-Sidak for multiple comparisons; (d), Mann-Whitney test; (e), (g), Kruskal-Wallis test with Dunn's multiple comparisons; (h), two-way ANOVA with Šidák correction; (j), Chi-square test; (l), (p), D'Agostino-Pearson normality test and unpaired t-test. $0.001 < {**}\ p < 0.01$; $0.01 < {*}\ p < 0.05$.

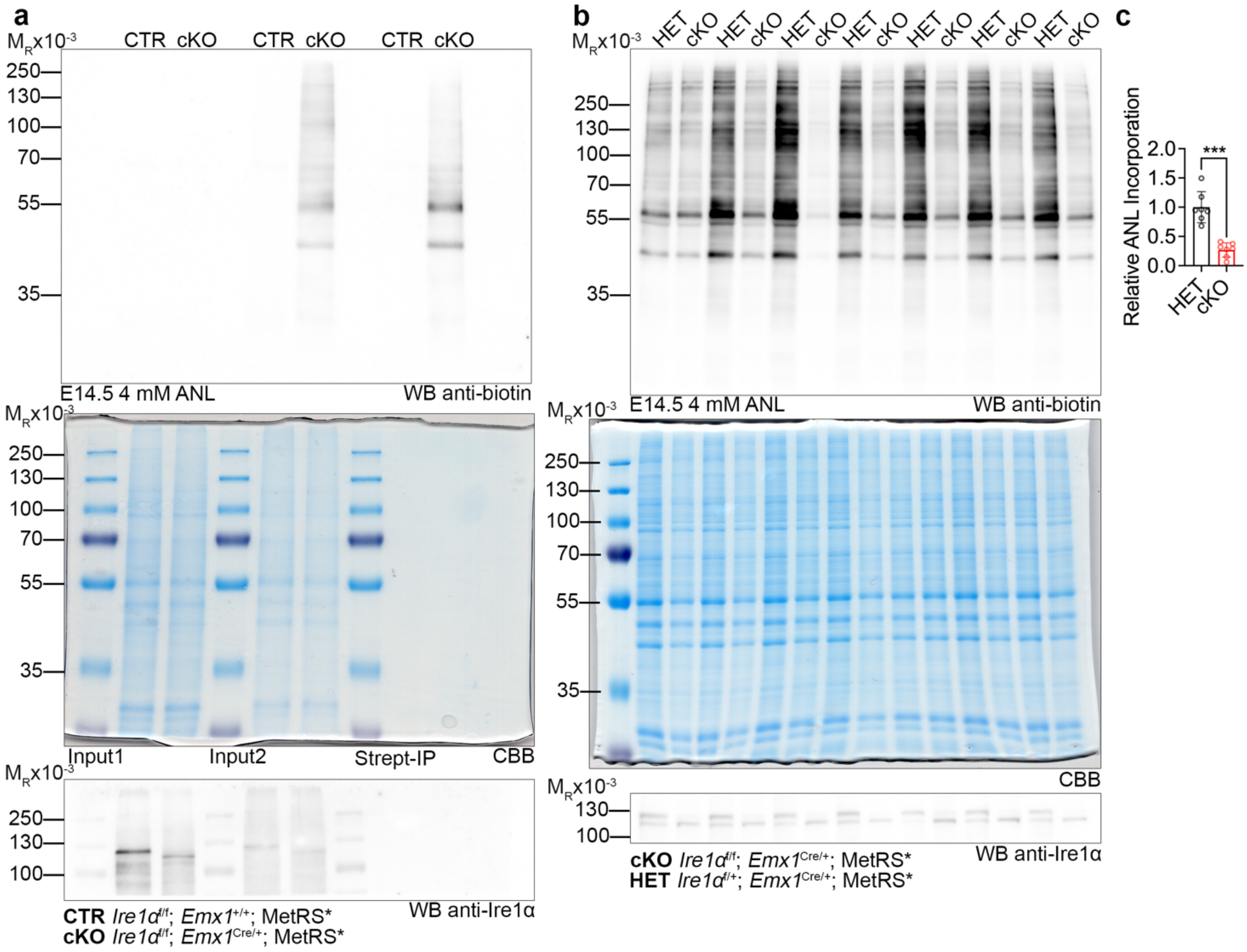

Fig. S10. Related to Fig. 8. Loss of Ire1α leads to diminished translation rates in the developing cortex.

(a) Representative results of Western blotting in E14.5 cortical lysates after L-azidonorleucine (ANL) exposure using indicated antibodies. Input 1 - before ANL-biotin click reaction in the homogenate, Input 2 - after the ANL-biotin click reaction and Strept-IP - after the immunoprecipitation of biotin using streptavidin beads. Coomassie brilliant blue, CBB. (b) Western blotting in cortical homogenates after ANL exposure and click reaction (Input 2). (c) Quantification of the ANL incorporation in the homogenate, normalized to the total protein input. Bar graphs indicate mean ± S.D. D'Agostino-Pearson normality test and Mann-Whitney test. *** $p < 0.001$.

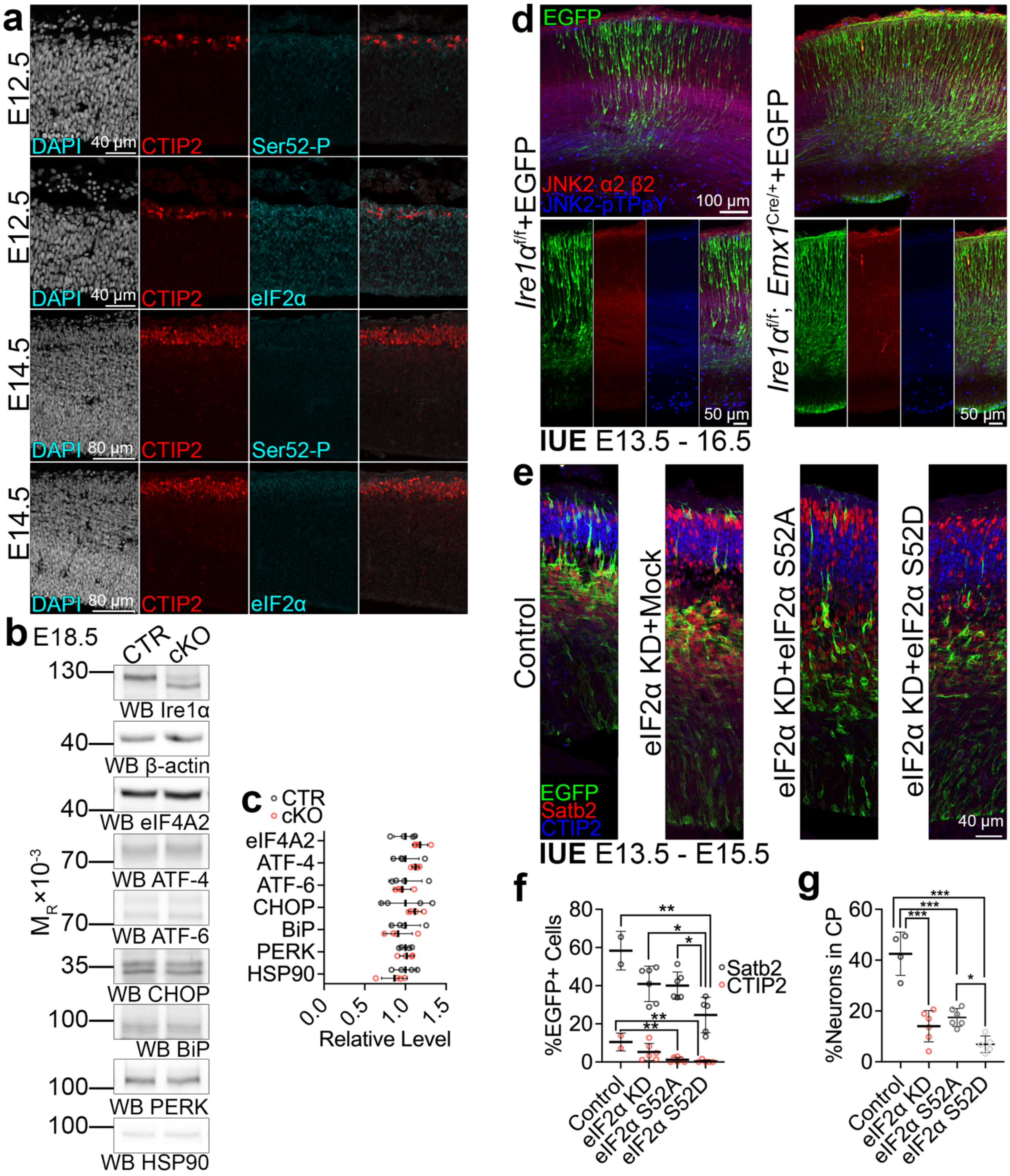

Fig. S11. Related to Fig. 8. Non-canonical role of Ire1α in the developing cortex.
(a) Representative immunostaining results in E12.5 and E14.5 cortices using anti-eIF2α and anti-eIF2α Ser52-P antibody. (b) Representative Western blotting results in control and cKO E18.5 cortex. (c) Quantification of protein levels from (b). (d) Representative results of immunostaining in the control and cKO cortex after IUE at E13.5. (e). Representative images of EGFP fluorescence signals and immunolabeling for Satb2 and CTIP in E15.5 brains of wild-type embryos after IUE at E13.5 with plasmids combinations encoding for EGFP, sh-RNA against eIF2α and phospho-deficient and phospho-mimetic variant of eIF2α. (f) Quantification

of neuronal identity and (g) cortical plate entry. Bar graphs indicate mean ± S.D. For statistics, (c), D'Agostino-Pearson normality test and unpaired t-test or Mann-Whitney test; (f-g), one-way ANOVA and Tukey multiple comparisons test. *** $p < 0.001$; $0.001 <$ ** $p < 0.01$; $0.01 <$ * $p < 0.05$.

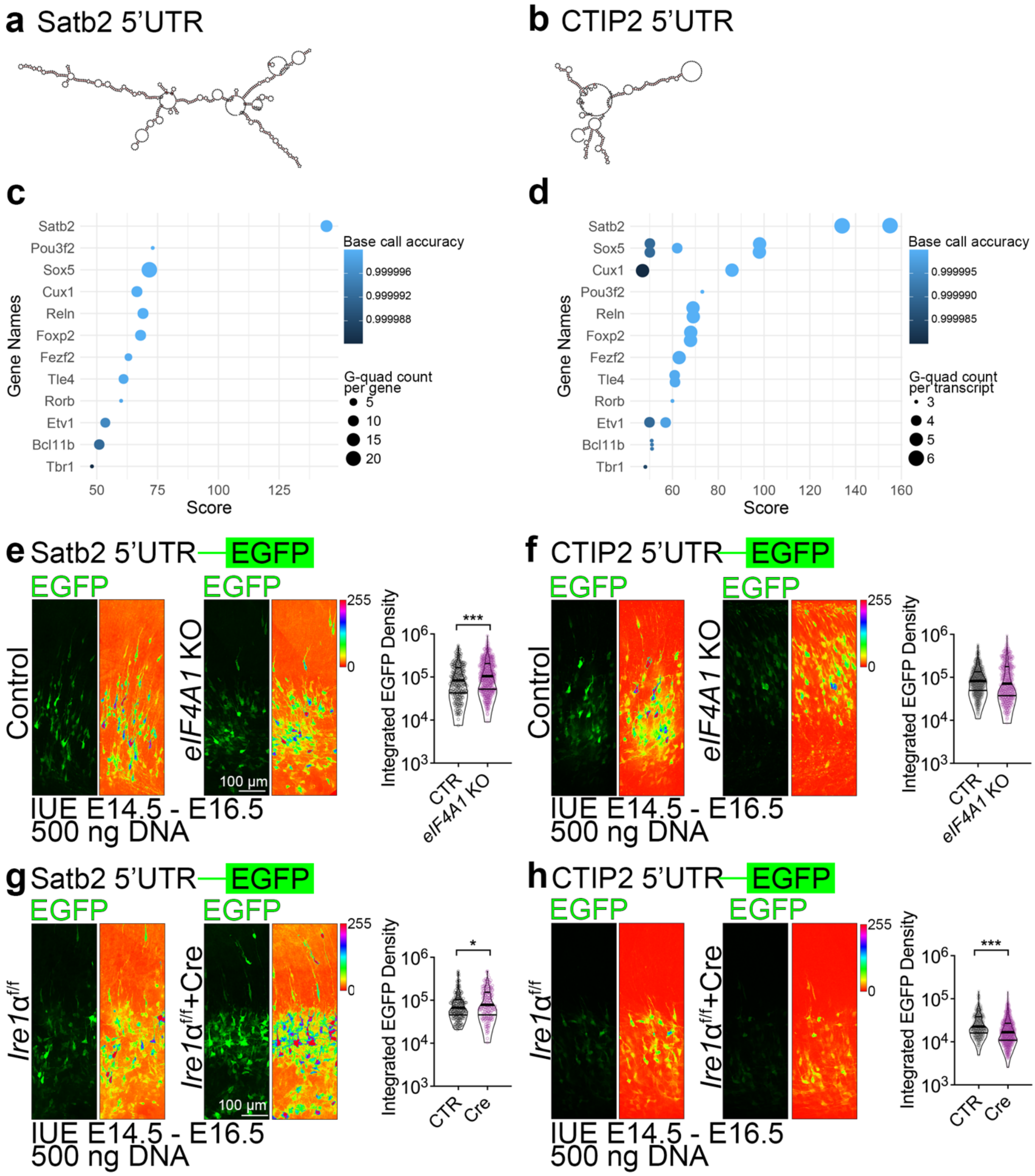

Fig. S12. Related to Fig. 9. eIF4A1- and Ire1α-driven translation regulation of cell identity.

(a-b) Models of 5'UTR mRNA structures of Satb2 and CTIP2. (c-d) Quantification of putative G-quadruplexes in indicated neuronal fate determinants. Plotted are the scores of sequence motif estimation per gene (c), representing the mean score of all transcripts for that gene and per transcript (d). The circle size represents the sum of the G-quadruplexes identified per transcript. (e-h) Representative images of EGFP fluorescence signals in the E16.5 brain sections after IUE at E14.5 with indicated vectors and Satb2 5'UTR (e, g) or CTIP2 5'UTR (f, h) translation reporter construct. Shown are the native signals (left panels) and intensity encoding (right panels). Quantification of EGFP fluorescence signals in single cells expressing translation reporters of Satb2 (e, g) and CTIP2 (f, h). Violin plots depict individual values, thick

line median and thin line quartiles. For statistical analyses, D’Agostino and Pearson normality test and Mann-Whitney test.

a Fig. 5n. IUE E12.5 - E16.5 Single Plane

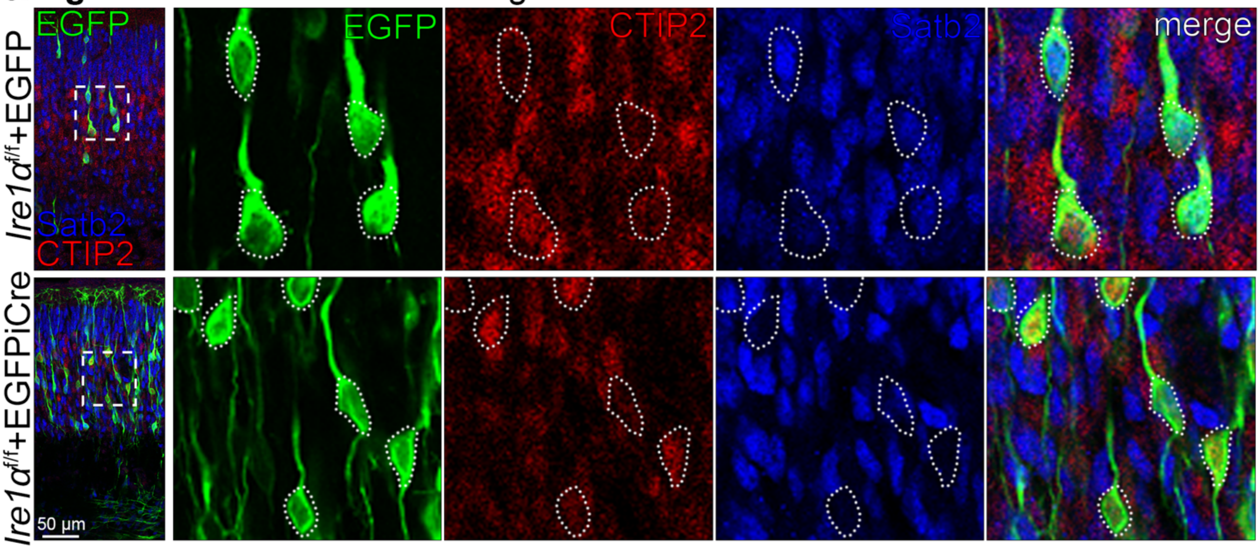

b Fig. 5p. IUE E12.5 - E18.5 Max Intensity Projection

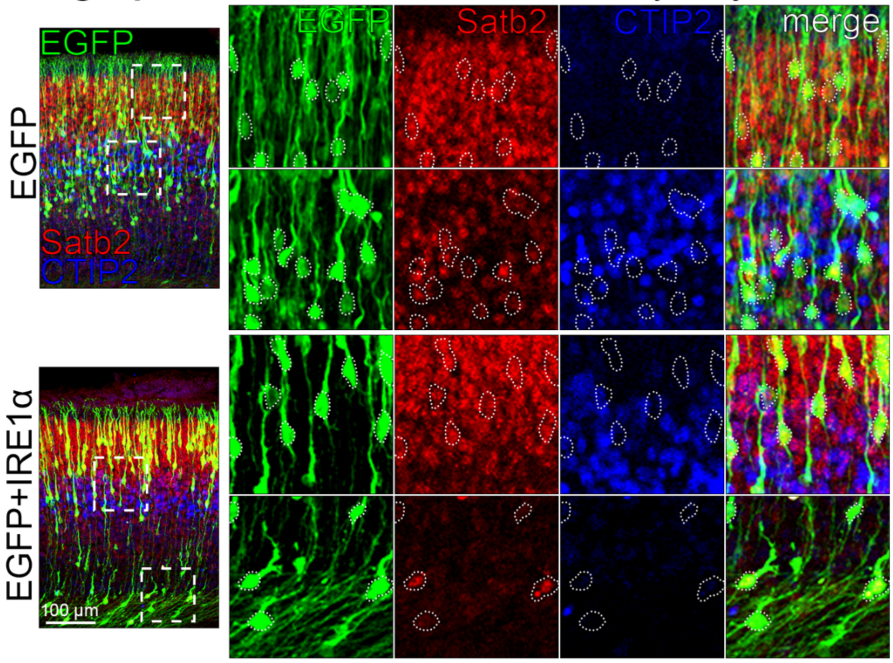

c Fig. 7e. IUE E12.5 - E18.5 Single Optical Plane

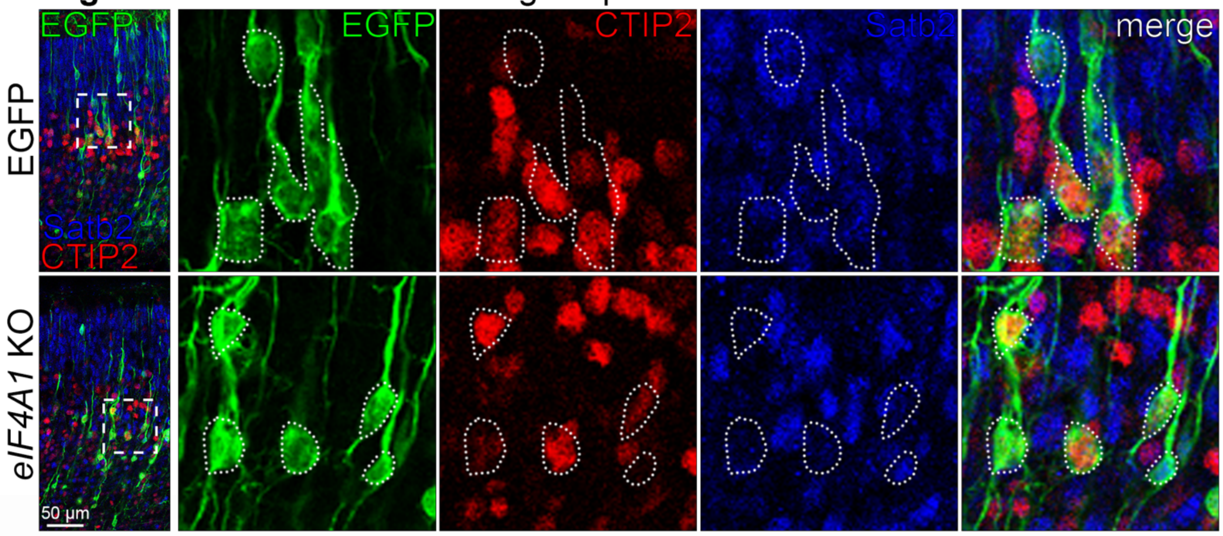

Fig. S13. Related to Fig. 5, Fig. 7. Zoom-ins for the neuronal identity phenotypes. (a-c) Representative images of immunostaining against EGFP, Satb2, and CTIP2 in coronal cortical sections after IUE at E12.5 for indicated conditions. Each panel represents a sample image pair used for identity analysis in experiment indicated on top. The squares depict the area of the zoom-in, outlined are somata of electroporated neurons. Neuronal identity was quantified in the proportion of all electroporated neurons in a given coronal section, here shown are snippets for a visualization of immunostaining.

Fig. 5h

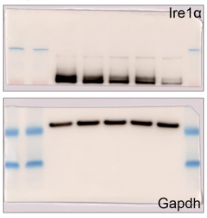

Fig. 5k

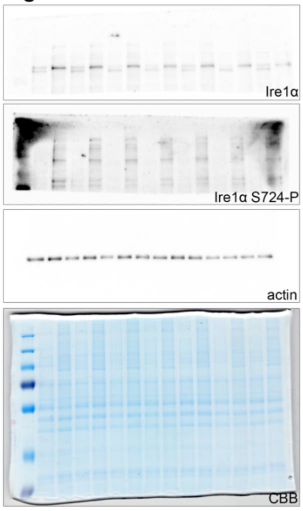

Fig. 7g+7i

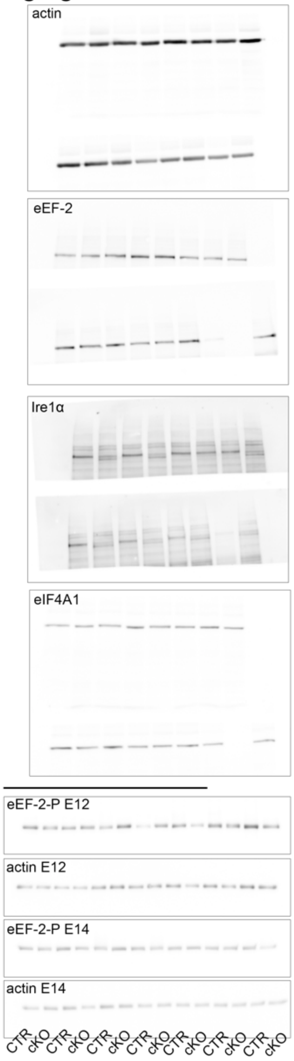

Fig. 7k

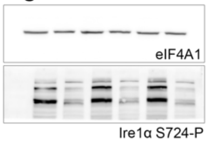

Fig. 7l

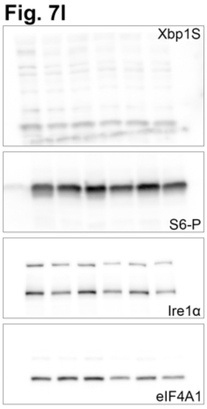

Fig. 7n

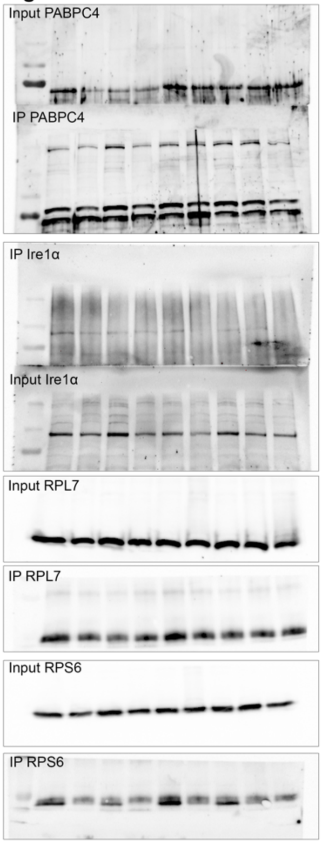

Fig. S3a

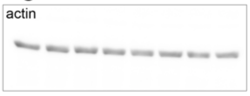

Fig. S9t

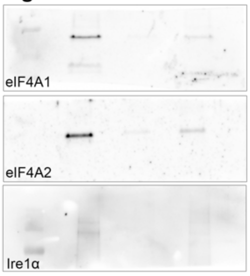

Fig. 7c+S9a

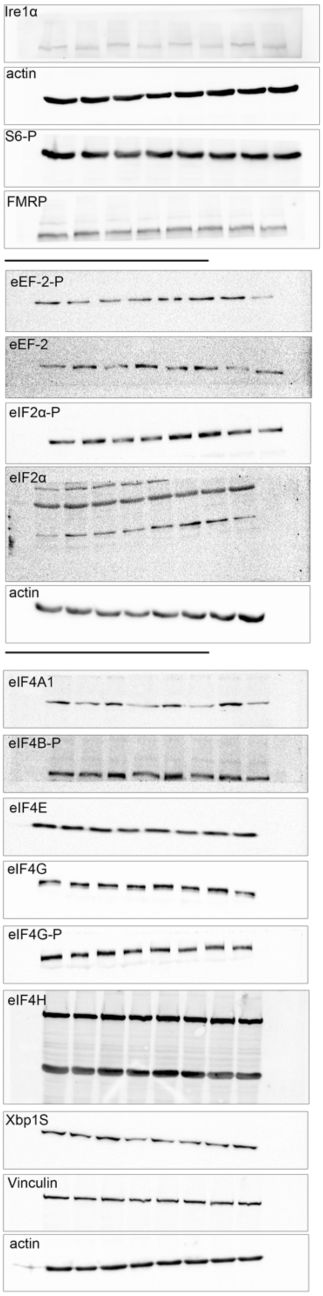

Fig. S9m

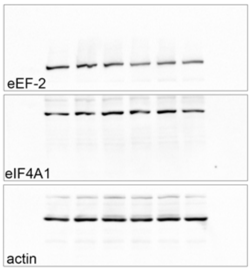

Fig. S9k

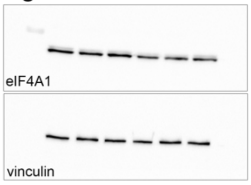

Fig. S10b

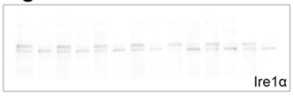

Fig. S11b

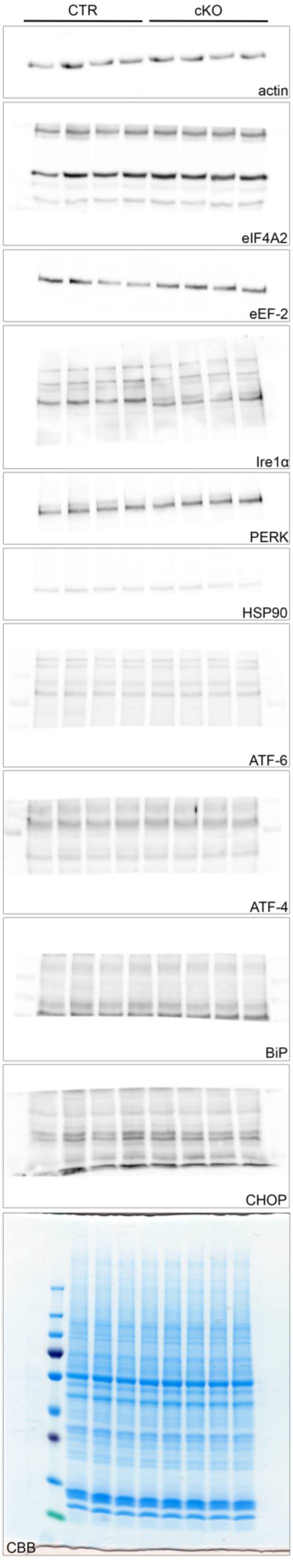

Fig. S14. Related to the entire manuscript. Pictures of Western blotting membranes used for the representative pictures in this manuscript.
Coomassie brilliant blue, CBB; a horizontal line separates experimental batches.
