# Supplementary material for: Protein translation rate determines neocortical neuron fate
Source: Nat Commun. 2024 Jun 7;15:4879. doi: 10.1038/s41467-024-49198-w (PMC11161512; doi:10.1038/s41467-024-49198-w)
Supplement: Supplementary file 3 — Description of Additional Supplementary Files [file 41467_2024_49198_MOESM3_ESM.pdf]

Description of Additional Supplementary Files

Supplementary Data S1. Numerical values, sample size and statistics for experiments in this paper.

Supplementary Data S2 Proteomics after puromycin pulse in E12.5 and E15.5 primary cortical cultures.

Supplementary Data S3. The results of the small inhibitor screening to identify regulators of Satb2.

Supplementary Data S4. Transcriptome analyses described in this study.

Supplementary Data S5. Key resources used in this work.
