# Supplementary material for: Protein translation rate determines neocortical neuron fate
Source: Nat Commun. 2024 Jun 7;15:4879. doi: 10.1038/s41467-024-49198-w (PMC11161512; doi:10.1038/s41467-024-49198-w)
Supplement: Supplementary file 9 — Reporting Summary [file 41467_2024_49198_MOESM9_ESM.pdf]

nature portfolio

Corresponding author(s): Mateusz C. Ambrozkiewicz

Last updated by author(s): May 16, 2024

Reporting Summary

Nature Portfolio wishes to improve the reproducibility of the work that we publish. This form provides structure for consistency and transparency in reporting. For further information on Nature Portfolio policies, see our Editorial Policies and the Editorial Policy Checklist.

Statistics

For all statistical analyses, confirm that the following items are present in the figure legend, table legend, main text, or Methods section.

n/a	Confirmed	
☐	☒	The exact sample size (*n*) for each experimental group/condition, given as a discrete number and unit of measurement
☐	☒	A statement on whether measurements were taken from distinct samples or whether the same sample was measured repeatedly
☐	☒	The statistical test(s) used AND whether they are one- or two-sided<br>*Only common tests should be described solely by name; describe more complex techniques in the Methods section.*
☐	☒	A description of all covariates tested
☐	☒	A description of any assumptions or corrections, such as tests of normality and adjustment for multiple comparisons
☐	☒	A full description of the statistical parameters including central tendency (e.g. means) or other basic estimates (e.g. regression coefficient) AND variation (e.g. standard deviation) or associated estimates of uncertainty (e.g. confidence intervals)
☐	☒	For null hypothesis testing, the test statistic (e.g. *F*, *t*, *r*) with confidence intervals, effect sizes, degrees of freedom and *P* value noted<br>*Give P values as exact values whenever suitable.*
☐	☒	For Bayesian analysis, information on the choice of priors and Markov chain Monte Carlo settings
☐	☒	For hierarchical and complex designs, identification of the appropriate level for tests and full reporting of outcomes
☐	☒	Estimates of effect sizes (e.g. Cohen's *d*, Pearson's *r*), indicating how they were calculated

Our web collection on statistics for biologists contains articles on many of the points above.

Software and code

Policy information about availability of computer code

Data collection	No commercial, open source, or custom code was utilized for data collection.
Data analysis	All code used in analysis of RNAseq can be found at https://github.com/qoldt/IRE1aKO_Polysome_RNAseq. We used the mouse genome assembly GRCm39 and gencode comprehensive gene annotation release M33. The genomic coordinates of the 5'UTR were extracted from the Genecode Release M12 (GRCm38.p5) reference genome obtained from the National Center for Biotechnology Information (NCBI). Code and software packages used in this study are detailed in the manuscript and include: MaxQuant software (v2.0.1.0), http://rna.tbi.univie.ac.at/cgi-bin/RNAWebSuite/RNAfold.cgi, pantherdb.org, pqsfinder, Salmon quant --validateMappings, ggplot2 package in R, gseaplot2 from the clusterProfilerpackage in R, emapplot from the enrichPlot package in R, DRIMseq, DESeq2, gseGO, Graph Pad Prism, Fiji.

For manuscripts utilizing custom algorithms or software that are central to the research but not yet described in published literature, software must be made available to editors and reviewers. We strongly encourage code deposition in a community repository (e.g. GitHub). See the Nature Portfolio guidelines for submitting code & software for further information.

Data

Policy information about availability of data

All manuscripts must include a data availability statement. This statement should provide the following information, where applicable:

- Accession codes, unique identifiers, or web links for publicly available datasets
- A description of any restrictions on data availability
- For clinical datasets or third party data, please ensure that the statement adheres to our policy

Sequencing data are available on NCBI under GSE172489. To review GEO accession GSE172489, go to https://www.ncbi.nlm.nih.gov/geo/query/acc.cgi?acc=GSE172489 and enter token gdsxiamgziginmn into the box. The mass spectrometry proteomics data have been deposited to the ProteomeXchange Consortium via the PRIDE partner repository with the dataset identifier PXD048919 (https://www.ebi.ac.uk/pride/archive?keyword=PXD048919). Source data are provided as a separate file and supplementary Fig. S13 and S14.

Research involving human participants, their data, or biological material

Policy information about studies with human participants or human data. See also policy information about sex, gender (identity/presentation), and sexual orientation and race, ethnicity and racism.

Reporting on sex and gender	n/a
Reporting on race, ethnicity, or other socially relevant groupings	n/a
Population characteristics	n/a
Recruitment	n/a
Ethics oversight	n/a

Note that full information on the approval of the study protocol must also be provided in the manuscript.

Field-specific reporting

Please select the one below that is the best fit for your research. If you are not sure, read the appropriate sections before making your selection.

☒ Life sciences ☐ Behavioural & social sciences ☐ Ecological, evolutionary & environmental sciences

For a reference copy of the document with all sections, see nature.com/documents/nr-reporting-summary-flat.pdf

Life sciences study design

All studies must disclose on these points even when the disclosure is negative.

Sample size	All detailed information on statistic, the exact numerical values and the sample size determination can be found in Supplementary Table S1. All reported replicates We did not predetermine the sample size. The sample size for our experiments were derived from our previous published studies and the standards in the field of sequencing and mass spectrometry according to practicality and financial feasibility.
Data exclusions	No data were excluded.
Replication	The number of replicates in our experiments are listed in Supplementary Table S1.
Randomization	Given the nature of labeling (IUE, transfection etc.) and nature of collected material (embryonic cortex), all samples randomly fall into analyzed categories. Embryonic and postnatal mice used in our experiments were utilized without sex distinction. Animals were randomly pooled across litters for the experiments. When pooling was required, this was also performed independent of the sex, per a biological replicate.
Blinding	Blinding was used in experiments requiring classification (axon numbers) and fluorescence measurements. For computational data analyses, we were not blinded to a sample identity.

Reporting for specific materials, systems and methods

We require information from authors about some types of materials, experimental systems and methods used in many studies. Here, indicate whether each material, system or method listed is relevant to your study. If you are not sure if a list item applies to your research, read the appropriate section before selecting a response.

Materials & experimental systems

n/a	Involved in the study	
☐	☒	Antibodies
☐	☒	Eukaryotic cell lines
☒	☐	Palaeontology and archaeology
☐	☒	Animals and other organisms
☒	☐	Clinical data
☒	☐	Dual use research of concern
☒	☐	Plants

Methods

n/a	Involved in the study	
☒	☐	ChIP-seq
☐	☒	Flow cytometry
☒	☐	MRI-based neuroimaging

Antibodies

Antibodies used	The detailed information on antibodies and other key resources used in this work can be found in the Methods section and Supplementary Table S5.
Validation	The validation of a key antibody used in this research can be found within this manuscript (e.g. Fig. 5, S9). For Satb2 antibody, we have previously validated it in our published works (Reference 21 and 38). Otherwise, we referred to the information provided by the commercial antibody suppliers.

Eukaryotic cell lines

Policy information about cell lines and Sex and Gender in Research

Cell line source(s)	Cell lines used in this study are primary cell lines from embryonic cortex or embryonic body wall (MEF). Given the nature of the preparation of the lines, the sex of the cells is mixed. HEK293T and NIH3T3 were from DSMZ (https://www.dsmz.de/).
Authentication	Authentication and characterization for neuronal cell cultures can be found on Fig. S1 and S2. For MEFs, morphology and growth dynamics were used to authenticate the cell line. Cell lines purchased from DSMZ were not authenticated.
Mycoplasma contamination	Primary cell lines were not tested for mycoplasma. HEK293T, Neuro-2A NIH3T3 were routinely negatively tested for the contamination using Mycoplasma Detection Assay (Eurofins).
Commonly misidentified lines (See ICLAC register)	n/a

Animals and other research organisms

Policy information about studies involving animals; ARRIVE guidelines recommended for reporting animal research, and Sex and Gender in Research

Laboratory animals	We used laboratory mice, Mus musculus, strains NMRI (Charles River), Emx1-Cre, Ire1af/f, Fucci2aR*, MetRS*, and Satb2-Cre mouse lines. Developmental stages or stages at experimental interventions are listed on the figures or in the figure legends.
Wild animals	n/a
Reporting on sex	Littermates of both sexes were randomly assigned to experimental groups during experimental procedures or collection of embryonic tissue. Developmental stages or stages at experimental interventions are listed on the figures or in the figure legends.
Field-collected samples	The study does not contain field-collected samples.
Ethics oversight	All experiments were performed in compliance with the guidelines for the welfare of experimental animals approved by the State Office for Health and Social Affairs, Council in Berlin, Landesamt für Gesundheit und Soziales (LaGeSo), permissions permissions G0079/11, G0206/16, G0184/20, G0054/19, G0055/19, T102/11 and T33/22, and by the Ethical Committee of the Lobachevsky State University of Nizhny Novgorod.

Note that full information on the approval of the study protocol must also be provided in the manuscript.

Plants

Seed stocks	n/a
Novel plant genotypes	n/a
Authentication	n/a

Flow Cytometry

Plots

Confirm that:

☒ The axis labels state the marker and fluorochrome used (e.g. CD4-FITC).

☒ The axis scales are clearly visible. Include numbers along axes only for bottom left plot of group (a 'group' is an analysis of identical markers).

☒ All plots are contour plots with outliers or pseudocolor plots.

☒ A numerical value for number of cells or percentage (with statistics) is provided.

Methodology

Sample preparation

Right after trituration, E13.5 embryonic cortical neurons prepared from Satb2Cre/+ mice, were nucleofected with pCAG-EGFP and pCAG-loxP-Stop-loxP-tdTomato plasmids and seeded at 120 000 cells per well of 96-well plate. Two hours post-plating, cultures were treated with compounds at two concentrations, in technical duplicates. Cells were then cultivated until DIV2, when the proportion of Satb2tdTom neurons normalized to EGFP positive cells was determined using FACS. Cells were washed with PBS, briefly incubated with 0.25% trypsin solution, followed by trituration in the presence of FBS before feeding into the instrument. Sample preparation description can also be found in PMID: 28782628.

The embryonic cerebral cortices were isolated and placed in ice-cold HBSS with MgCl2 and CaCl2 (HBSS+/+, Gibco). Before trypsinization, brains were washed by centrifuging at 600 rpm for 1 minute. Then, the supernatant was removed and replaced with 5 ml of fresh HBSS+/+. The tissue was digested by adding 500 µl of 2.5 % trypsin, 0.02 mg/ml DNase, and 1 µl of 25U/µl benzonase to 5 ml of HBSS +/+ and following incubation for 20 min at 37°C. The reaction was stopped by adding 2 ml of FBS. The samples were washed three times with FACS buffer (2% FCS, 0.02 mg/ml DNase, 1 µl of 25U/µl benzonase). Next, the cortices were carefully triturated in 300 µl of FACS buffer. Subsequently, samples were transported on ice in FACS buffer to the cell sorting facility. Cell sorting was performed by the BIH Cytometry Core Facility on a BD FACSAria Fusion (BD Biosciences, San Jose, CA, USA), configured with 5 lasers (UV, violet, blue, yellow-green, red). Samples were collected after sorting in 1.5 ml of FACS buffer. The cells were then centrifuged for 15 minutes at 1000 rpm. The samples were subsequently frozen and stored at -80°C until further use.

Instrument	BD FACSCanto™ II and BD FACSAria Fusion
Software	BD FACSDiva™ Software
Cell population abundance	The cells expressing EGFP and tdTomato were quantified in the post-sort fraction based on the measured fluorescence.
Gating strategy	FSC/SSC gates, as well as the cutoff values for EGFP, tdTomato expression and FT label were set prior to the screening experiments with control, untreated neurons. Gating was then unchanged for all tested compunds. The procedure is described in Ambrozkiewicz et al., J Neurosci Meth, 2017.

☒ Tick this box to confirm that a figure exemplifying the gating strategy is provided in the Supplementary Information.
